# Gain of function of TMEM16E/ANO5 scrambling activity caused by a mutation associated with gnathodiaphyseal dysplasia

**DOI:** 10.1007/s00018-017-2704-9

**Published:** 2017-11-09

**Authors:** Eleonora Di Zanni, Antonella Gradogna, Joachim Scholz-Starke, Anna Boccaccio

**Affiliations:** Institute of Biophysics, Consiglio Nazionale delle Ricerche, Via de Marini 6, 16149 Genova, Italy

**Keywords:** TMEM16E, Anoctamin5, Anoctamins, Phospholipid scramblase, Calcium-activated chloride channels, Phosphatidylserine

## Abstract

Mutations in the human *TMEM16E* (*ANO5*) gene are associated both with the bone disease gnathodiaphyseal dysplasia (GDD; OMIM: 166260) and muscle dystrophies (OMIM: 611307, 613319). However, the physiological function of TMEM16E has remained unclear. We show here that human TMEM16E, when overexpressed in mammalian cell lines, displayed partial plasma membrane localization and gave rise to phospholipid scrambling (PLS) as well as non-selective ionic currents with slow time-dependent activation at highly depolarized membrane potentials. While the activity of wild-type TMEM16E depended on elevated cytosolic Ca^2+^ levels, a mutant form carrying the GDD-causing T513I substitution showed PLS and large time-dependent ion currents even at low cytosolic Ca^2+^ concentrations. Contrarily, mutation of the homologous position in the Ca^2+^-activated Cl^−^ channel TMEM16B paralog hardly affected its function. In summary, these data provide the first direct demonstration of Ca^2+^-dependent PLS activity for TMEM16E and suggest a gain-of-function phenotype related to a GDD mutation.

**Electronic supplementary material:**

The online version of this article (doi:10.1007/s00018-017-2704-9) contains supplementary material, which is available to authorized users.

## Introduction

Gnathodiaphyseal dysplasia (GDD; OMIM: 166260) is a rare autosomal-dominant generalized skeletal syndrome characterized by fibro-osseous lesions of the jawbones and associated with long and tubular bone dysplasia and fragility. GDD patients show facial deformity, begin to experience frequent bone fractures around puberty and are susceptible to purulent osteomyelitis in jawbones during adult life [1, 28]. This syndrome has been associated to mutations in the *GDD1* gene [36], also known as *TMEM16E* or *Ano5*, encoding a 913-amino acid integral membrane protein of unknown physiological function. At present, eight GDD-causing *TMEM16E* mutations have been identified leading to amino acid exchanges at six positions: p.Arg215Gly [17], p.Cys356Gly, p.Cys356Arg [36], p.Cys356Tyr [2, 9, 17, 38], p.Cys360Tyr [17], p.Ser500Phe [29], p.Thr513Ile [22] and p.Gly518Glu [17].

In humans and mice, the *TMEM16E* gene is highly expressed in skeletal muscle and bone tissues, such as calvaria, femur and mandibule [36]. In particular, it is expressed in human osteoblasts and periodontal ligament cells, consistent with GDD disease phenotypes [36]. Unlike other TMEM16 family members, TMEM16E does not show a clear plasma membrane (PM) localization, at most weakly [23], but rather in unspecified intracellular vesicles [23, 34]. When heterologously overexpressed, it was found predominantly localized in the ER network [10, 13, 36] or at the PM [34].

TMEM16E belongs to a family of integral membrane proteins named TMEM16 or anoctamins [16, 26]. According to the recently published crystal structure of the fungal homologue nhTMEM16 [6], TMEM16 proteins have 10 transmembrane domains. Although they share a similar membrane topology, they can perform diverse cellular functions: TMEM16A and TMEM16B function as Ca^2+^-activated chloride channels, while TMEM16F acts as a Ca^2+^-dependent phospholipid scramblase facilitating the movement (scrambling) of phospholipids between the leaflets of the membrane bilayer. There is no such consensus about the functional roles of other family members.

Available data on TMEM16E function are few and not conclusive. Attempts to directly record electrical activity of heterologously expressed TMEM16E failed [10, 35] or revealed small Ca^2+^-activated currents [34], and no anion transport activity was detected in halide-sensitive YFP-based fluorescence assays [22, 31]. Based on amino acid conservation between family members, Tran et al. [35] inserted the GDD-related C356G and C356R exchanges [36] at the corresponding position in the PM-localized TMEM16A protein. Only the protein carrying the glycine exchange gave rise to currents, which, quite surprisingly, appeared to display cationic selectivity, as opposed to anionic in wild-type TMEM16A [35]. Due to the predominant intracellular localization of TMEM16E, several studies have furthermore relied on chimeric constructs based on the TMEM16A backbone. A chimeric protein carrying the putative TMEM16E channel pore region was detected at the PM, although not conductive [35], while three different chimeras created by Duran et al. [10] were retained intracellularly. Recently, following the identification of a specific 35-aa-long scrambling domain necessary and sufficient for TMEM16F activity [41], Gyobu et al. [13, 14] measured scrambling activity of a chimeric protein carrying the homologous region of TMEM16E in the TMEM16A backbone, indicating that the 35-aa stretch worked as a scrambling domain. Scramblase activity for the TMEM16E wild-type protein still awaits confirmation.

Here, we find partial PM localization of heterologously expressed TMEM16E, which allowed us to perform functional studies on the ion transport and scrambling activity of TMEM16E. Furthermore, we identify a gain-of-function phenotype for a *TMEM16E* mutation related to gnathodiaphyseal dysplasia.

## Results

### TMEM16E-EGFP fusion proteins show partial plasma membrane localization

**Fig. 1:**
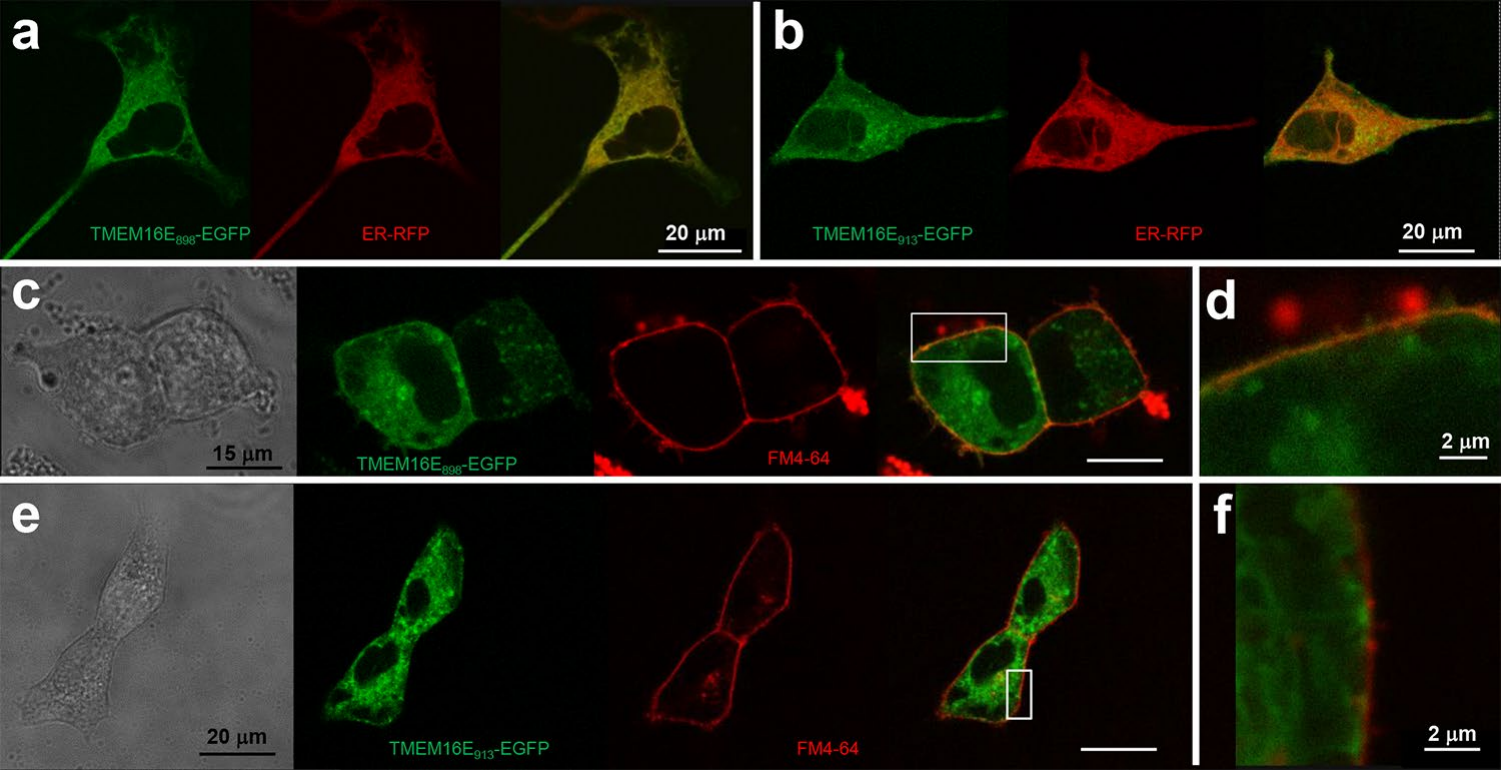
Localization of TMEM16E-EGFP fusion proteins. **a**, **b** Confocal images of HEK293 cells transfected with the TMEM16E_898_-EGFP (**a**) or TMEM16E_913_-EGFP (**b**) fusion constructs and co-stained with the ER marker CellLight ER-RFP. Left, green channel (EGFP); middle, red channel (ER-RFP); right, merge. **c–f** Co-staining of HEK293 cells expressing TMEM16E_898_-EGFP (**c**, **d**) or TMEM16E_913_-EGFP (**e**, **f**) with the PM marker FM4-64. From left to right: in transmission light, in the green channel (EGFP), in the red channel (FM4-64), merge of green and red channels. **d**, **f** Close-up views of the squared regions indicated in **c**, **e**

The 898-aa-long TMEM16E isoform derived from Saos-2 cells [22] was transiently overexpressed as an EGFP fusion protein in HEK293 cells (TMEM16E_898_-EGFP; Fig. 1a). In accordance with previous studies [10, 13], EGFP-tagged TMEM16E localized mostly to intracellular membranes, showing significant overlap with the ER marker CellLight ER-RFP (Fig. 1a). In cells displaying high expression levels, however, EGFP signals partially lined the cell boundaries and co-localized with the PM marker FM4-64 (Fig. 1c, d). A similar pattern was found in cells transiently expressing the full-length (913-aa-long) TMEM16E isoform, although the PM localization was less evident in this case (Fig. 1b, e, f).

### TMEM16E mediates Ca^2+^-dependent non-selective ionic currents

**Fig. 2:**
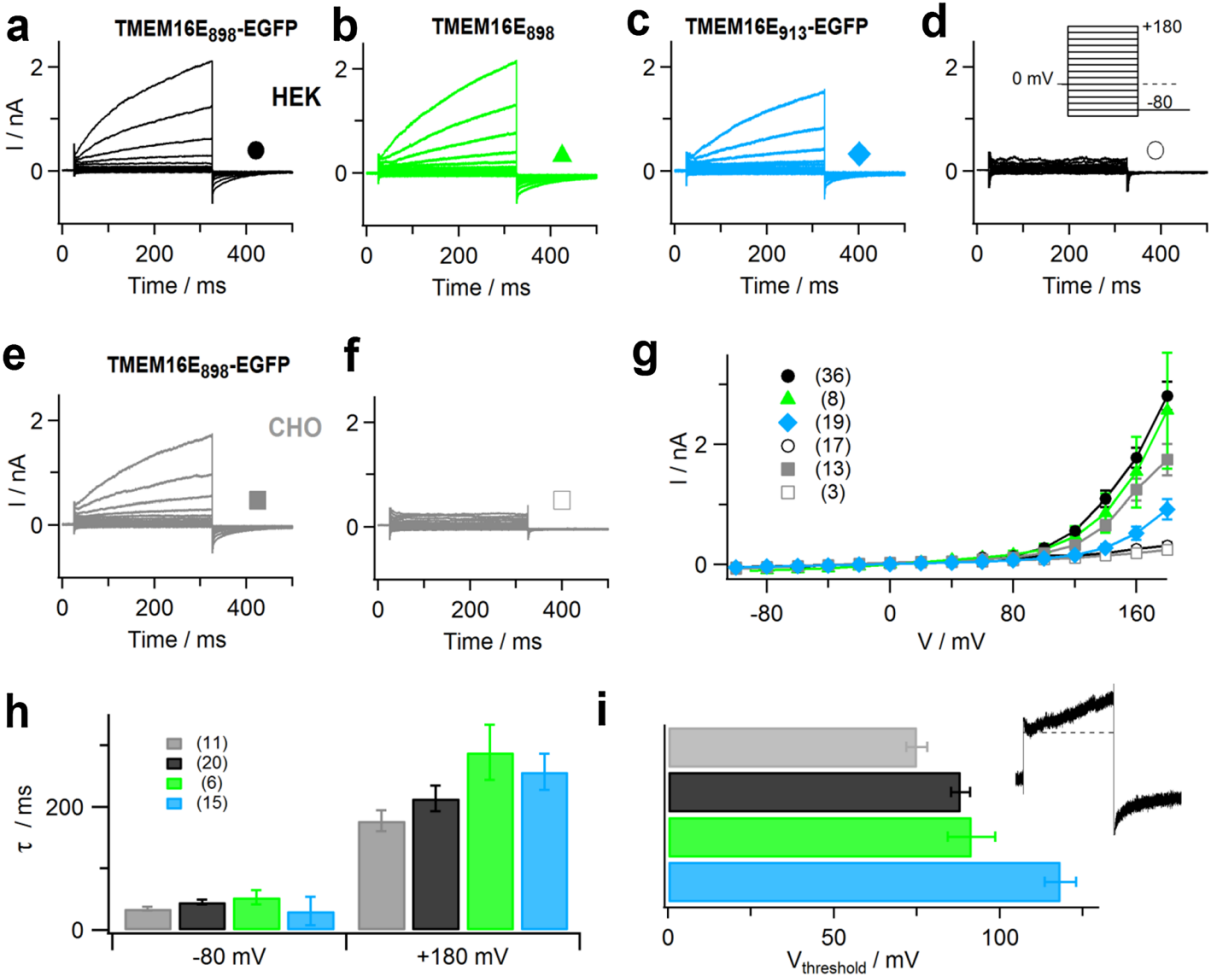
Ion transport activity of TMEM16E proteins. **a–f** Whole-cell patch-clamp recordings with standard intracellular solution containing 3 µM calculated free Ca^2+^, in HEK293 cells transfected with TMEM16E_898_-EGFP (**a**), TMEM16E_898_ (**b**), TMEM16E_913_-EGFP (**c**) and in a non-transfected HEK293 cell (**d**); in a CHO cell transfected with TMEM16E_898_-EGFP (**e**) and a non-transfected CHO cell (**f**). The stimulation protocol (inset in **d**) consisted of 300-ms voltage steps ranging from − 100 to + 180 mV with 20-mV increments, followed by a 175-ms tail pulse to − 80 mV. Holding potential at 0 mV. **g** Average I–V relationships in HEK293 and CHO cells, non-transfected and transfected with TMEM16E-EGFP constructs. Symbols as indicated in **a**–**f**, *n* given in brackets. **h** Average relaxation time constants (*τ*) of membrane currents are plotted versus the applied membrane potential. Colors as indicated in **a**–**c, e**, *n* given in brackets. **i** Average threshold potentials (*V*_threshold_) of TMEM16E current activation, for TMEM16E_898_-EGFP in CHO (*n* = 8; gray bar) and TMEM16E_898_-EGFP (*n* = 31; black bar), TMEM16E_898_ (*n* = 7; green bar), TMEM16E_913_-EGFP (*n* = 18; blue bar) in HEK293. Inset: current traces illustrating the first membrane potential at which time-dependent currents were observed, defined as *V*_threshold_. Error bars indicate sem in all panels

Subsequently, we conducted whole-cell patch-clamp experiments on HEK293 cells transfected with plasmid constructs encoding TMEM16E_898_-EGFP (Fig. 2a), untagged TMEM16E_898_ (Fig. 2b) or full-length TMEM16E_913_-EGFP (Fig. 2c). In the presence of 3 μM calculated free intracellular Ca^2+^, these recordings showed large, time-dependent outward currents at membrane potentials higher than about + 100 mV, shortly (< 2 min) after the establishment of the whole-cell configuration. Similar currents were also observed in CHO cells transiently expressing TMEM16E_898_-EGFP (Fig. 2e). Average current amplitudes were comparable for the TMEM16E_898_ constructs expressed in HEK293 and CHO cells, yet significantly lower for the full-length TMEM16E_913_ isoform (Fig. 2g), which was consistent with its weaker PM targeting (Fig. 1e, f). Under identical experimental conditions, non-transfected control cells presented only small background currents lacking any time dependence (Fig. 2d, f, g). In particular, outward currents possibly due to endogenous TMEM16F expression in HEK293 cells [32] were absent for up to 20 min in the whole-cell configuration (data not shown). The kinetics of current activation and deactivation were similar for the different TMEM16E constructs (Fig. 2h), suggesting that the cellular expression system and the presence of the EGFP tag had only minor effects on the basic ion transport properties of TMEM16E.

Since TMEM16E ion currents activated at relatively high positive membrane potentials and did not reach saturation within the applied voltage range, we evaluated current activation from the threshold potential **(** *V*_threshold_; Fig. 2i), defined as the first potential step at which time-dependent currents were observed, as illustrated in the inset. Average *V*_threshold_ values were + 88.1 ± 2.8 mV (*n* = 31) for TMEM16E_898_-EGFP, + 91.4 ± 7.2 mV (*n* = 7) for untagged TMEM16E_898_, both expressed in HEK293 cells, and + 75.0 ± 3.1 mV (*n* = 8) for TMEM16E_898_-EGFP expressed in CHO cells. The construct TMEM16E_913_-EGFP (in HEK293 cells) showed more positive values (+ 118.3 ± 4.7 mV, *n* = 18), possibly related to its lower PM expression and current amplitudes, which limits the accuracy of threshold determination.

**Fig. 3:**
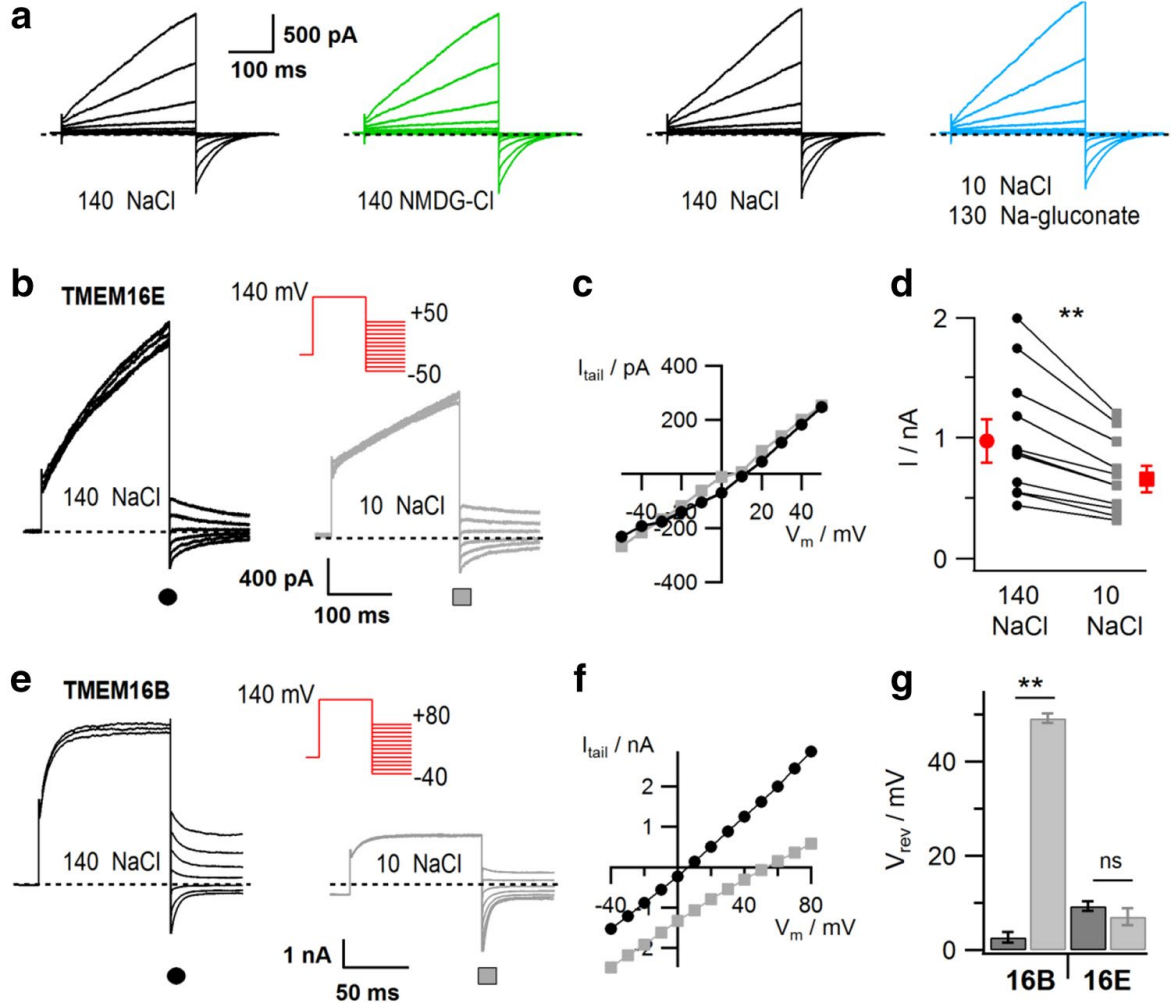
Ionic selectivity of TMEM16E-mediated currents. **a** Whole-cell patch-clamp recordings in a HEK293 cell transfected with TMEM16E_898_-EGFP, successively exposed to standard bath solution (140 NaCl), NMDG-Cl and Na-gluconate solution, as indicated. Stimulation protocol as in the inset of Fig. 2d. **b** Tail current recordings in a HEK293 cell transfected with TMEM16E_898_-EGFP, successively exposed to standard bath solution (140 mM NaCl; left traces) and bath solution containing 10 mM NaCl (right traces). The stimulation protocol (inset) consisted of a prepulse to + 140 mV followed by voltage steps between − 50 and + 50 mV with 10-mV increments. For clarity, only current traces in 20-mV steps are shown. **c** I–V relationships of the tail current recordings shown in **b**. **d** Current amplitudes at the +140 mV prepulse in 140 mM NaCl and 10 mM NaCl bath solution, for individual cells (*n* = 11) and average (red symbols; paired *t* test, *P* = 7.9 × 10^−4^). **e** Tail current recordings as in **b**, but in a HEK293 cell transfected with TMEM16B-EGFP and using a stimulation protocol (inset) with voltage steps between − 40 and + 80 mV with 10-mV increments. **f** I–V relationships of the tail current recordings shown in **e**. **g** Average reversal potentials (*V*_rev_) of tail current recordings in 140 mM NaCl (dark gray bars) and 10 mM NaCl bath solution (light gray bars), for TMEM16B-EGFP (*n* = 6; paired *t* test, **P = 2 × 10^−7^) and TMEM16E_898_-EGFP (*n* = 8; paired *t* test, *P* = 0.14, ns not significant). Error bars indicate sem in all panels

**Fig. 4:**
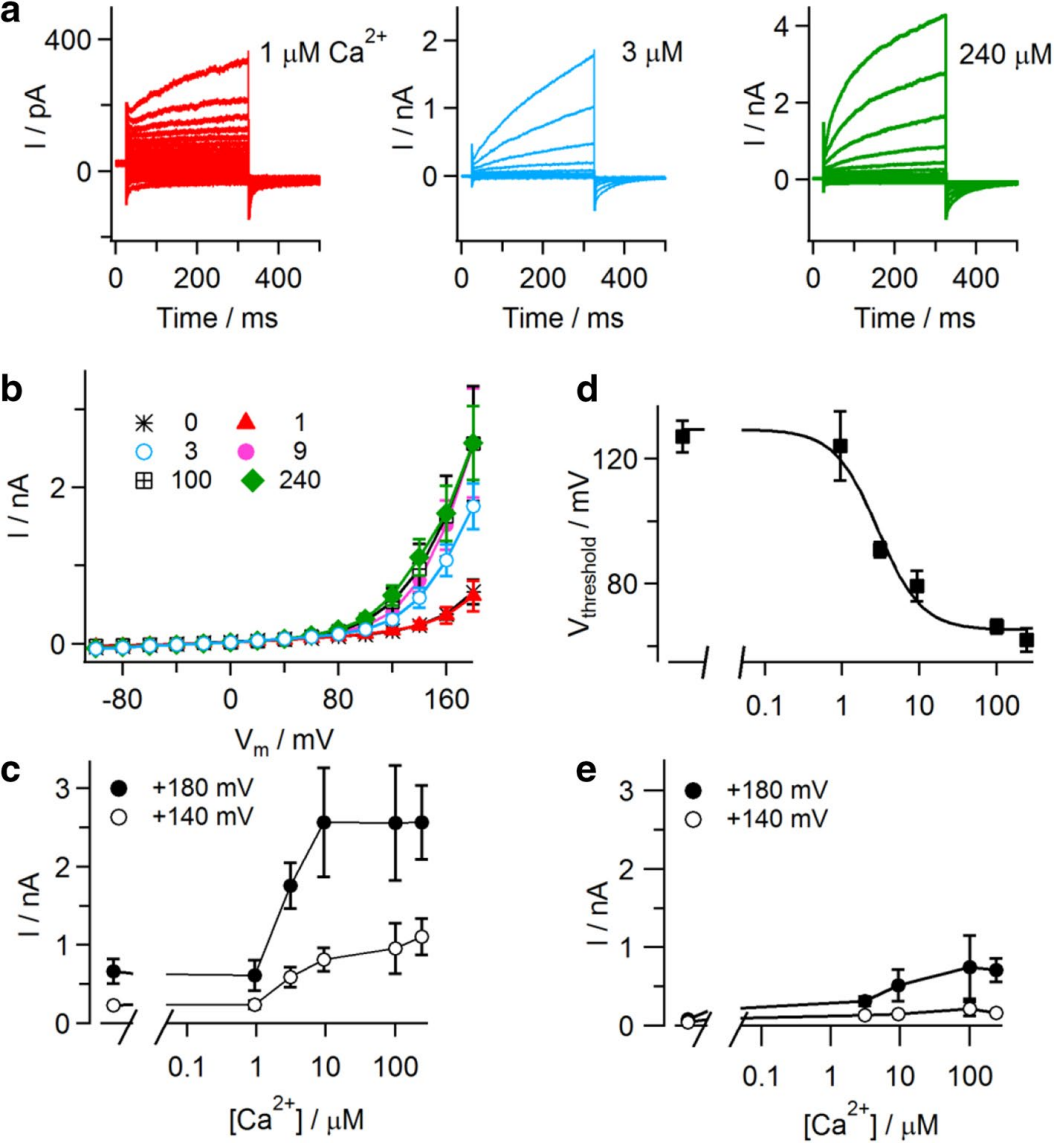
Calcium dependence of TMEM16E-mediated ionic currents. **a** Whole-cell patch-clamp recordings in CHO cells transfected with TMEM16E_898_-EGFP, in the presence of 1 µM (left), 3 µM (middle) and 240 µM (right) calculated free Ca^2+^ in the intracellular solution. Note the different current amplitude scales. Stimulation protocol as in the inset of Fig. 2d. **b** Average I–V relationships derived from current recordings at different intracellular free Ca^2+^ concentrations, as partly shown in **a**. **c** Ca^2+^ dependence of current amplitudes recorded at + 140 and + 180 mV (data from **b**). **d** Threshold potentials (*V*_threshold_) of TMEM16E current activation are plotted as a function of the intracellular free Ca^2+^ concentration (*n* = 7 for zero Ca^2+^, 9, 100, 240 µM; *n* = 5 for 1 µM; *n* = 10 for 3 µM). Data points were fitted with a Hill function (continuous line) yielding a half-maximal concentration of 2.9 µM Ca^2+^ and a Hill coefficient of 1.5. **e** Average current amplitudes recorded at different intracellular free Ca^2+^ concentrations in non-transfected CHO cells. Error bars indicate sem in all panels

TMEM16E currents showed little change in their amplitude, when Na^+^ and Cl^−^ in the bath solution were exchanged for the large ions NMDG and gluconate (Fig. 3a), suggesting that TMEM16E is highly non-selective and its permeation pathway relatively large, similarly to what has been observed for TMEM16F [41]. In order to investigate ion selectivity in more detail, we reduced the external NaCl concentration from 140 to 10 mM (adjusting the osmolarity with sucrose) and determined the reversal potential (*V*_rev_) of TMEM16E currents by applying a tail protocol (Fig. 3b, inset). At low NaCl, we observed a reduction of the pre-pulse current amplitude (Fig. 3b, d; ratio 0.69 ± 0.02, *n* = 11), however no change of *V*_rev_ (Fig. 3b, c, g). The moderate amplitude reduction is also consistent with a low ion selectivity, as it indicates that other ions in the bath or pipette solution drive the residual current in the presence of low NaCl. By contrast, currents mediated by the Ca^2+^-activated Cl^−^ channel TMEM16B displayed robust anion/cation selectivity (Fig. 3e–g, [4]). The pre-pulse current reduction of TMEM16B (Fig. 3e) is due to reduced channel activation in low chloride, as reported by Betto et al. [4].

Current recordings in CHO cells expressing TMEM16E_898_-EGFP revealed that the presence of cytosolic Ca^2+^ was not strictly necessary for TMEM16E activation, but strongly favored it. Average outward current amplitudes were small both at zero Ca^2+^ and at 1 μM free Ca^2+^, progressively increased at concentrations up to 10 μM and finally saturated at higher concentrations up to 240 μM (Fig. 4a–c). In non-transfected CHO cells, appreciable background currents were only present at the highest Ca^2+^ concentrations and the most extreme positive membrane potentials (Fig. 4e). For TMEM16E-mediated currents, the threshold potential (see Fig. 2i) showed a clear dependence on the cytosolic Ca^2+^ concentration, shifting from 127 ± 5 mV (*n* = 7) at zero Ca^2+^ to 62 ± 4 mV at 240 μM Ca^2+^ (*n* = 7; Fig. 4d and Supplementary Figure 2). Fitting the data points with a Hill function yielded a half-maximal Ca^2+^ concentration of 2.9 µM and a Hill coefficient of 1.5.

### TMEM16E exhibits Ca^2+^-dependent phospholipid scramblase activity

**Fig. 5:**
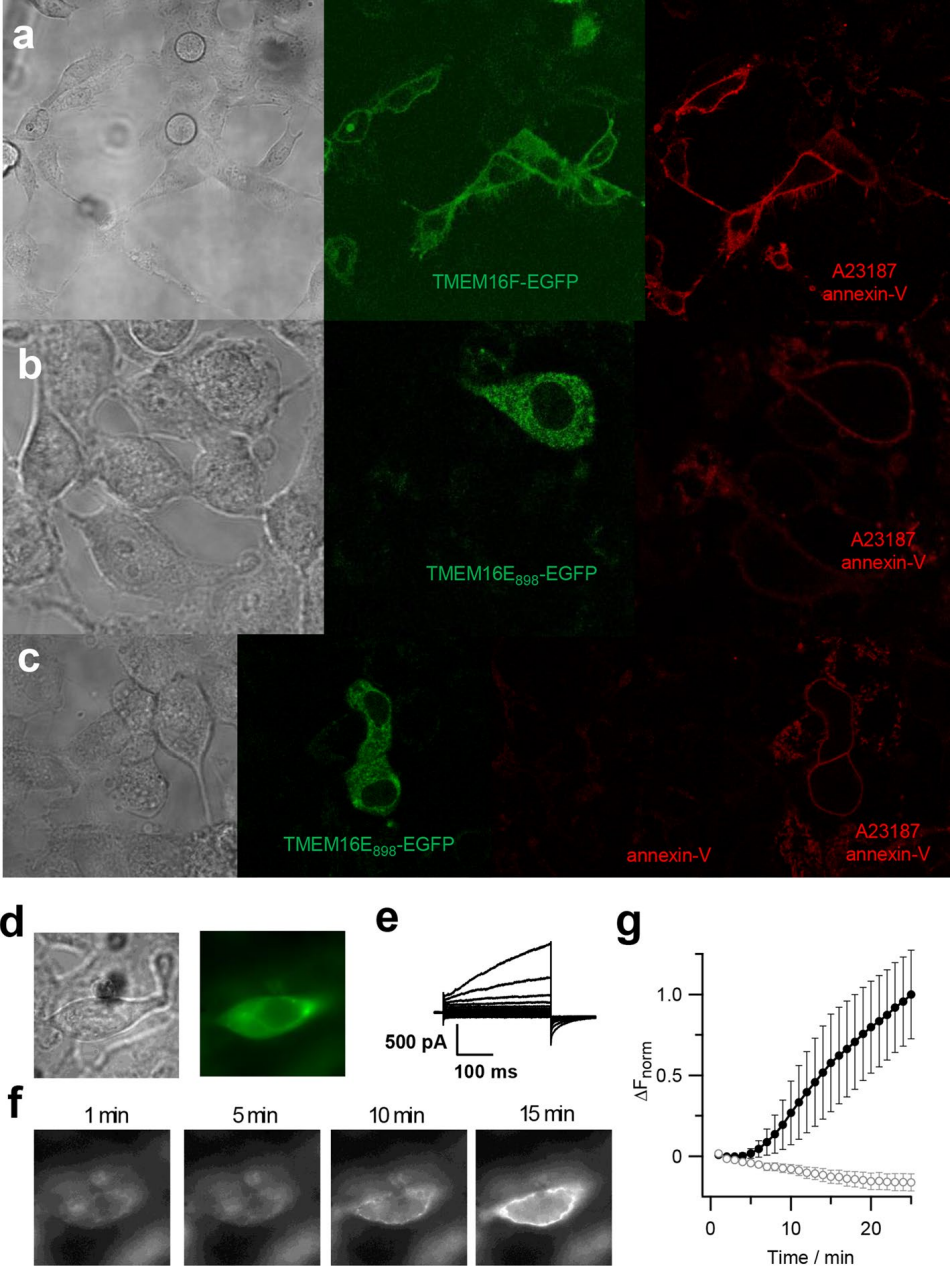
Phospholipid scrambling activity of TMEM16E. **a–c** Confocal images of live HEK293 cells transfected with TMEM16F-EGFP (**a**) or TMEM16E_898_-EGFP (**b**, **c**) and treated with the Ca^2+^ ionophore A23187 (5 µM) for 4 min, in the presence of 5 mM extracellular Ca^2+^ and Cy3-conjugated annexin-V. From left to right, transmission light, green channel (EGFP), red channel (Cy3). In **c**, Cy3 fluorescence before and after A23187 treatment. **d–g** Activation of PLS activity in whole-cell patch-clamp recordings. In HEK293 cells expressing TMEM16E_898_-EGFP (**d**), membrane currents were elicited immediately after reaching the whole-cell configuration, applying voltage steps from − 100 to + 140 mV in 20-mV increments, from a holding potential of 0 mV (**e**). Fluorescence images (**f**) were recorded at the indicated whole-cell time points, with 3 μM free Ca^2+^ in the patch pipette and Alexa Fluor 555-conjugated annexin-V in the bath solution. **g** Average normalized fluorescence intensity change (∆*F*_norm_) of recordings shown in **f**, after background fluorescence subtraction, for TMEM16E_898_-EGFP expressing cells (*n* = 10; filled circles) and non-transfected control cells (*n* = 4; open circles). Error bars indicate sem

Next, we investigated the phospholipid scrambling (PLS) activity of TMEM16E using an annexin-V binding assay on HEK293 cells expressing TMEM16E_898_-EGFP. TMEM16F-EGFP was used as a positive control (Fig. 5a). When extracellular Ca^2+^ entry was stimulated with the Ca^2+^ ionophore A23187 in the presence of annexin-V (conjugated to Cy3 dye), we observed clear fluorescence signals lining the cell boundaries of EGFP-positive cells after 5–10 min, but not of non-transfected neighboring cells (Fig. 5b, c). No annexin-V binding was observed in the absence of Ca^2+^ ionophore (Fig. 5c). Both with TMEM16E and TMEM16F, not all transfected cells were marked by annexin-V, possibly due to insufficient Ca^2+^ entry. However, we avoided stronger stimuli since they could induce apoptosis, thereby evoking phosphatidylserine exposure in all cells.

We further performed patch-clamp experiments on TMEM16E_898_-EGFP transfected HEK293 cells, simultaneously recording ionic currents and imaging PLS activity in the presence of Alexa Fluor 555-conjugated annexin-V in the bath solution (Fig. 5d–g). TMEM16E ion currents became apparent quickly after reaching the whole-cell configuration, activated by 3 μM free Ca^2+^ in the intracellular solution entering the cytoplasm (Fig. 5e). Currents mediated by TMEM16E remained relatively stable over time, although we noted a time-dependent increase of background currents in these recording conditions (Supplementary Figure 3), possibly partly due to a modification of the membrane properties by PLS activity and/or extracellularly bound annexin-V. Fluorescence signals lining the cell boundaries, indicative of annexin-V binding, became detectable on average after 5.2 ± 0.7 min (*n* = 10) in the whole-cell configuration and strongly increased over time (Fig. 5f, g). A lag phase of similar length between current activation and PLS detection was also reported for TMEM16F [41]. In non-transfected control cells, no time-dependent currents developed and no annexin-V binding was observed within the 25-min recording period (Fig. 5g and Supplementary Figure 3c).

### The GDD-causing T513I substitution affects TMEM16E scrambling and ion transport activities

**Fig. 6:**
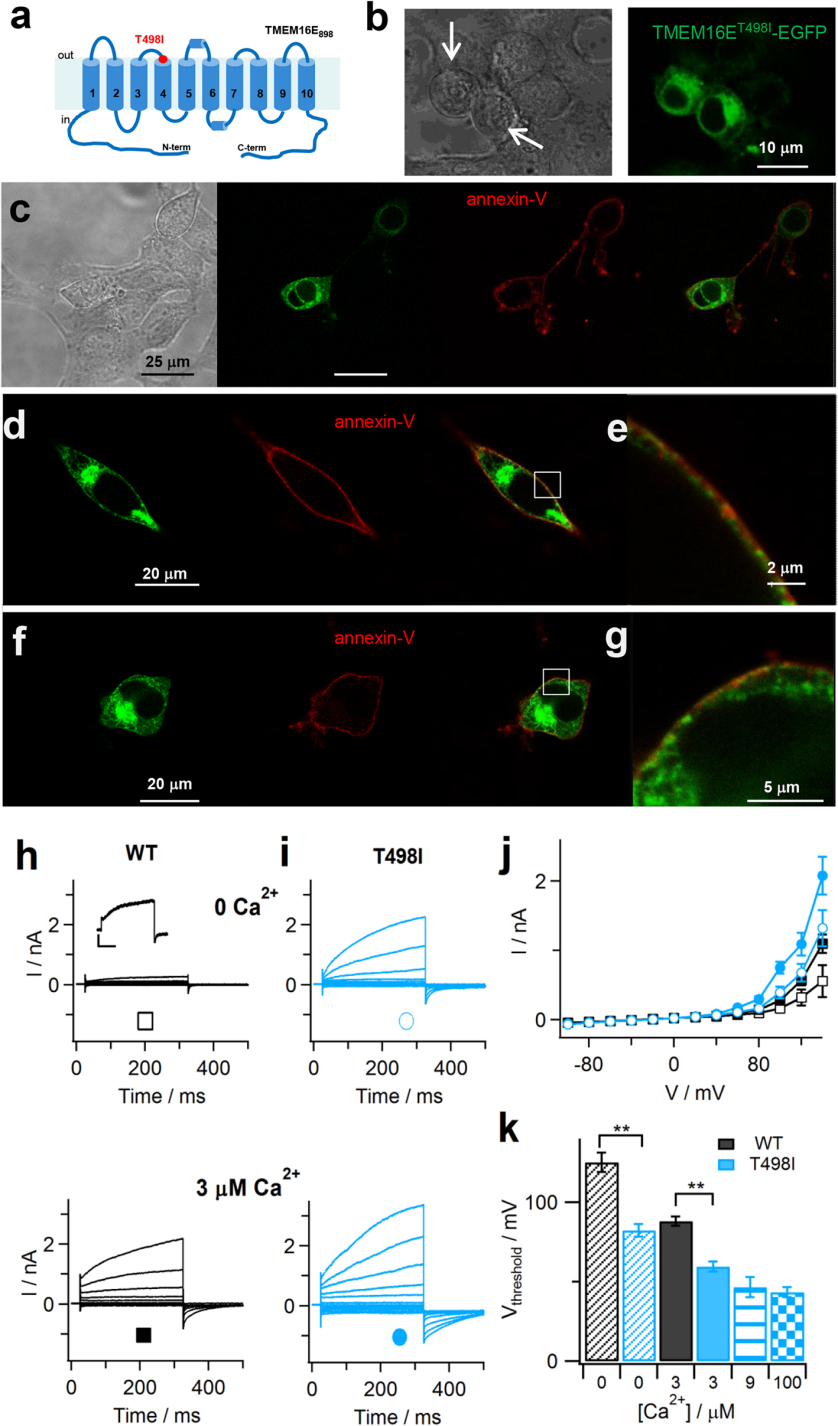
TMEM16E gain-of-function caused by the GDD-related T513I substitution. **a** Putative TMEM16E membrane topology, based on nhTMEM16 and mTMEM16A protein structures [6, 25]. The position of the T513I amino acid exchange, corresponding to T498I in TMEM16E_898_, is indicated. **b** Confocal images of HEK293 cells transfected with TMEM16E_898_^T498I^-EGFP showing impaired cell adhesion and round shape (arrows). Left, transmission light; right, green channel (EGFP). **c–g** Cy3-conjugated annexin-V binding to HEK293 cells transfected with TMEM16E_898_^T498I^-EGFP in the absence of Ca^2+^ ionophore. Live cell sample in **c**, fixed samples in **d**, **f**. From left to right: in transmission light, in the green channel (EGFP), in the red channel (Cy3), merge of green and red channels. **e**, **g** Close-up views of the squared regions indicated in **d**, **f**. **h**, **i** Whole-cell patch-clamp recordings in HEK293 cells expressing WT (**h**) or T498I mutant (**i**) TMEM16E_898_-EGFP, with intracellular solutions containing zero Ca^2+^ (upper traces) or 3 µM free Ca^2+^ (lower traces). The stimulation protocol consisted in 300-ms voltage steps ranging from − 100 to + 140 mV with 20-mV increments, followed by a 175-ms tail pulse to − 80 mV. Inset in **h**: current traces showing time-dependent currents at + 140 mV. Scale bars, 100 pA/100 ms. **j** Average I–V relationships derived from recordings as shown in **h**, **i**, for WT (*n* = 18 at zero Ca^2+^; *n* = 35 at 3 µM) and T498I mutant protein (*n* = 11 at zero Ca^2+^; *n* = 17 at 3 µM). **k** Threshold potentials (*V*_threshold_) of TMEM16E current activation at zero Ca^2+^ (*n* = 19 WT, *n* = 11 T498I), 3 µM (*n* = 31 WT, *n* = 12 T498I), 9 µM (*n* = 9 T498I) and 100 µM free Ca^2+^ (*n* = 12 T498I). Mann–Whitney *U* test, **P = 2 × 10^−5^ at zero Ca^2+^ and 3 × 10^−7^ at 3 µM Ca^2+^. Error bars indicate sem in all panels

We investigated the effect of the GDD-causing T513I substitution [22], located in the second extracellular loop (Fig. 6a), on TMEM16E localization and function by introducing it into the TMEM16E_898_-EGFP construct (T498I; Table 1 and Supplementary Figure 1). In striking contrast to wild-type TMEM16E (Fig. 1), HEK293 cells expressing the T498I mutant construct, at > 48 h post-transfection, typically became round-shaped and displayed decreased cell adhesion (Fig. 6b, arrows), similarly to what has been observed in COS-7 cells expressing the TMEM16E^C356G^ and TMEM16E^C356R^ mutants [36]. Between 36 and 48 h after transfection, however, most cells displayed a normal morphology and the TMEM16E^T498I^-EGFP mutant protein showed WT-like localization, as testified by the partial overlap between the EGFP and Cy3 fluorescence signals at the PM in annexin-V binding assays (Fig. 6e, g).

**Table 1:** Numeration of isoforms and the GDD-causing T513I substitution in the TMEM16 proteins used in this study

	TMEM16E_913_	TMEM16E_898_	TMEM16B
Origin	Human	Human	Mouse
Total length (aa)	913	898	913
Position of substitution	T513	T498	T489

*aa* amino acids

Surprisingly, these assays showed that annexin-V bound to cell membranes even in the absence of Ca^2+^ ionophore (Fig. 6c–g), indicating that PLS activity of the TMEM16E^T498I^ mutant protein was no longer dependent on elevated cytosolic Ca^2+^ concentrations. Patch-clamp recordings fully confirmed this result: differently from the wild-type protein (Fig. 6h, j), TMEM16E^T498I^ mediated large time-dependent currents even at zero Ca^2+^, which further increased in amplitude at 3 μM free Ca^2+^ in the pipette solution (Fig. 6i, j). The threshold potential of TMEM16E^T498I^ activation shifted from + 82.3 ± 4.1 mV at zero Ca^2+^ (*n* = 11) to + 59.6 ± 3.2 mV at 3 μM Ca^2+^ (*n* = 12), while the respective *V*_threshold_ values for TMEM16E^WT^ were + 125.2 ± 6.1 mV (*n* = 19) and + 88.1 ± 2.8 mV (*n* = 31; Fig. 6k), almost identical to the WT data determined in CHO cells (Fig. 4d). *V*_threshold_ at saturating [Ca^2+^] was around + 43 mV for the T498I mutant (Fig. 6k), compared to + 62 mV for the WT protein, indicative of a significant shift of the Ca^2+^ dependence towards more negative membrane potentials. Thus, the data of both current amplitudes and threshold potentials concurrently demonstrate that, as a consequence, TMEM16E^T498I^ activity at zero Ca^2+^ is comparable to TMEM16E^WT^ activity at 3 μM cytosolic Ca^2+^, strongly suggesting that the GDD-causing T513I substitution causes a gain-of-function phenotype for the TMEM16E protein.

### Minor effects of the T513I substitution introduced into TMEM16B

**Fig. 7:**
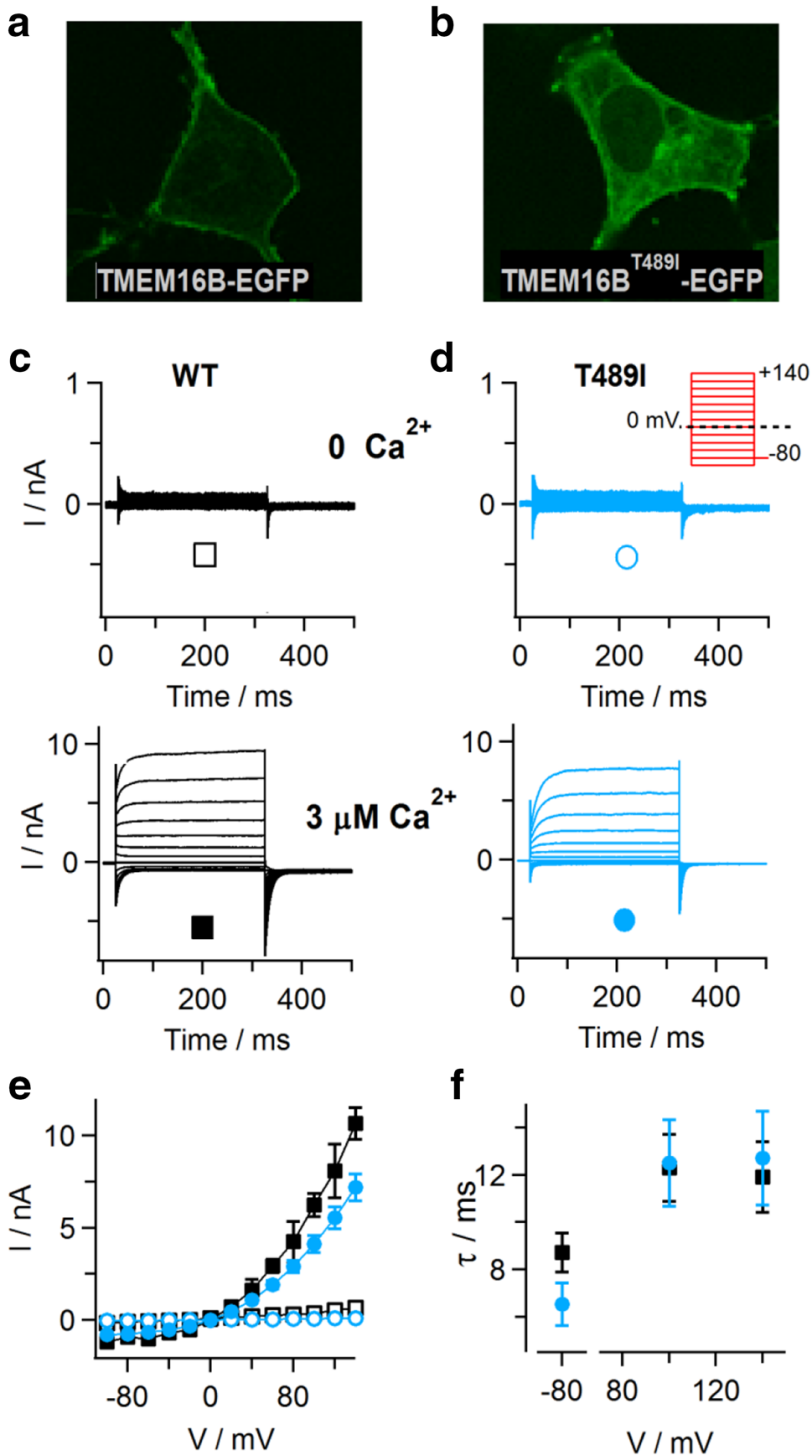
Expression of TMEM16B carrying the GDD-related T489I substitution. **a**, **b** Confocal images of HEK293 cells transiently expressing TMEM16B-EGFP (**a**) and TMEM16B^T489I^-EGFP (**b**). **c**, **d** Whole-cell patch-clamp recordings with standard intracellular solution containing zero Ca^2+^ (upper traces) or 3 µM free Ca^2+^ (lower traces), in HEK293 cells expressing TMEM16B-EGFP (**c**) and TMEM16B^T489I^-EGFP (**d**). Inset in **d**, stimulation protocol. **e** Average steady-state I–V relationships of recordings as shown in **c**, **d**, for WT (*n* = 6 in each condition) and T489I mutant protein (*n* = 7 at zero Ca^2+^, *n* = 19 at 3 µM Ca^2+^). **f** Relaxation time constants (*τ*) of currents recorded at 3 µM free Ca^2+^ are plotted versus the applied membrane potential, for WT (*n* = 6) and T489I mutant protein (*n* = 16). Symbols in **e**, **f** as indicated in **c**, **d**. Error bars indicate sem in all panels

The position T513 is not strictly conserved between TMEM16E orthologs of different vertebrate species [22], some presenting an alanine residue at this position. However, a threonine residue is present and conserved in TMEM16 family members working as Ca^2+^-activated Cl^−^ channels, TMEM16A and TMEM16B. Therefore, we introduced the T513I substitution at the homologous position of TMEM16B (T489I; Table 1 and Supplementary Figure 1), in order to evaluate its effect on protein localization and function of this well-characterized Ca^2+^-activated Cl^−^ channel. Wild-type TMEM16B-EGFP fusion protein was preferentially targeted to the PM in transiently transfected HEK293 cells (Fig. 7a). By contrast, although EGFP fluorescence signals in cells expressing the TMEM16B^T489I^ mutant similarly lined the cell borders, there was additionally strong intracellular staining (Fig. 7b). Whole-cell patch-clamp recordings showed that, similarly to TMEM16B^WT^ currents, time-dependent outward currents in TMEM16B^T489I^-expressing cells were absent at zero Ca^2+^ in the pipette solution (Fig. 7c, d). Likewise, the current amplitudes recorded at 3 μM Ca^2+^ (Fig. 7e) as well as the kinetics of current activation and deactivation (Fig. 7f) were comparable to WT values. Taken together, although the T489I substitution caused increased intracellular retention, the TMEM16B^T489I^ protein conserved WT-like PM expression and ion channel function, suggesting that the amino acid position corresponding to T513 in TMEM16E has divergent roles in phospholipid scrambling and chloride channel activity of TMEM16 family members.

## Discussion

This study provides direct evidence for phospholipid scrambling and non-selective ion transport activity of TMEM16E, a member of the TMEM16 protein family [26] which is functionally split into Ca^2+^-activated Cl^−^ channels (TMEM16A/B) and Ca^2+^-activated phospholipid scramblases (TMEM16C/D/F/G/J). The identification of TMEM16E function represents a crucial step towards the definition of its physiological role, especially since mutations in the human *TMEM16E* gene are related to severe genetic diseases.

The prevalently intracellular localization of TMEM16E [10, 23] has significantly delayed the clarification of its function. In accordance with previous reports [10, 13, 36], TMEM16E-EGFP fusion proteins expressed in HEK293 and CHO cells showed strong co-localization with a ER marker; however, in cells with high expression levels, we additionally observed partial PM targeting. PM localization of heterologously overexpressed TMEM16E has also been reported by Tian et al. [34]. Moreover, in patch-clamp recordings, they found a significant increase in the membrane conductance (measured in the limited voltage range of ± 50 mV) upon application of the Ca^2+^ ionophore ionomycin. The results presented here provide the first detailed characterization of the kinetics, voltage- and Ca^2+^ dependence and ion selectivity of TMEM16E-mediated ion currents. Compared to previous studies which failed in this regard [10, 34, 35], a combination of factors may have contributed, among which the TMEM16E protein isoform, the expression vector, the time window of optimal expression and, importantly, the exploration of a wider range of positive membrane potentials.

Ionic currents mediated by TMEM16E were strikingly similar to those observed for TMEM16F [32, 41], the closest paralogue within the mammalian TMEM16 protein family. They were strongly outward rectifying, activating at highly depolarized membrane potentials, showed slow kinetics of activation and deactivation and were poorly ion selective. For both proteins, ionic currents displayed half-maximal activation at cytosolic [Ca^2+^] in the low micromolar range, although the Ca^2+^ dependence appears slightly less steep for TMEM16E [32]. Furthermore, contrarily to TMEM16F [32], TMEM16E showed low basal ion transport activity at highly depolarized potentials in the absence of cytosolic Ca^2+^. It is also noteworthy that TMEM16E-mediated currents were observed immediately after the establishment of the whole-cell configuration (even at low [Ca^2+^]), differently to TMEM16F-dependent currents, which consistently presented an activation delay of several minutes [12, 18, 32, 33, 41], indicative of negative regulation by a putative cytosolic factor in heterologous expression systems.

Importantly, we show here that, alike TMEM16F [32, 41], also TMEM16E has phospholipid scrambling activity in transiently transfected HEK293 cells. The crystal structure of the fungal nhTMEM16 lipid scramblase shows a lateral hydrophilic furrow facing the lipid bilayer, which appears to accommodate the hydrophilic head groups of phospholipids during their translocation across the membrane [6]. This hydrophilic furrow is thought to provide also an unspecific pathway for the ion transport observed in TMEM16F, consistent with its highly non-selective nature [41]. Similarly, the two fungal homologs nhTMEM16 and afTMEM16 show both lipid and non-selective ion transport activity [19, 21]. Based on similarities to TMEM16F currents, we propose the same origin for TMEM16E-mediated currents. Considering furthermore their exclusive activation at highly depolarized membrane potentials, which are unlikely to be experienced by non-excitable cells, one may conclude that ion transport is not among the physiological functions of TMEM16E. It constitutes, nevertheless, a reliable real-time readout of TMEM16E activity, for example in studies of its Ca^2+^ dependence and structure–function relationships.

Our data collectively support the idea of TMEM16E working as a Ca^2+^-activated phospholipid scramblase. This finding is an important step towards the identification of its physiological role, but detailed information about its subcellular localization in bone and skeletal muscle cells is now absolutely essential, in order to understand where and under which physiological circumstances its scrambling activity is required. The ability of TMEM16E to carry out PLS has been anticipated by a recent study in which the TMEM16E region corresponding to the 35-aa-long scrambling domain, identified in TMEM16F [41], was introduced into the TMEM16A backbone [13, 14]. Our data provide the direct demonstration of PLS mediated by the wild-type TMEM16E protein.

The determination of TMEM16E function will be equally instrumental to clarify its involvement in genetic diseases. Mutations in the human *TMEM16E* gene have been associated both with the autosomal-dominant skeletal dysplasia GDD and with two different forms of recessively inherited muscular dystrophy, proximal LGMD2L (limb-girdle muscular dystrophy-2 l; OMIM: 611307, [15, 20] and Miyoshi myopathy, OMIM: 613319, [5]), consistent with the highest *TMEM16E* expression found in bone and skeletal muscle [23, 36, 37]. To date, more than 70 different *TMEM16E* mutations have been reported in muscular dystrophy patients [11] and are believed to cause a loss-of-function phenotype, also since some of them are non-sense mutations leading to a truncated protein. Contrarily, based on the type of inheritance and the absence of overlap with muscular dystrophies, GDD-associated mutations may cause a gain of function. The data presented here provide the first direct evidence in favor of this hypothesis: the TMEM16E mutant protein carrying the T513I substitution [22], while preserving the subcellular localization of the wild-type protein, both mediated PLS that was no longer dependent on elevated cytosolic Ca^2+^ levels and carried large outward currents even at extremely low intracellular Ca^2+^ concentrations. These data strongly suggest that constitutive TMEM16E scrambling activity at basal cytosolic Ca^2+^ levels may lead to the pathological consequences observed in the bone tissue of GDD patients.

Despite poor sequence homology among TMEM16 family members in the extracellular loop connecting transmembrane domain 3 and 4, threonine 513 is conserved in the PM-localized Ca^2+^-activated Cl^−^ channels TMEM16A and TMEM16B. Substitution of the corresponding position in TMEM16B caused partial intracellular retention, but no major changes in the functional properties and Ca^2+^ dependence of the mutant protein. This result indicates that, during evolution, the second extracellular loop harboring the T513 residue and two further residues affected in GDD (S500F and G518E; [17, 29] has adopted differential roles in Cl^−^ channel members within the TMEM16 family compared to phospholipid scramblases.

In summary, we exploited the partial plasma membrane localization of heterologously overexpressed TMEM16E (1) to directly demonstrate phospholipid scrambling and non-selective ion transport activity of this elusive member of the TMEM16 family and (2) to identify a gain-of-function phenotype for the T513I substitution related to the autosomal-dominant skeletal dysplasia GDD. These results pave the way for the identification of its physiological role and the functional characterization of further TMEM16E mutants related to GDD and muscular dystrophies.

## Materials and methods

### DNA constructs, cell cultures and transfection

The cDNA clone of human TMEM16E, isolated from the Saos-2 osteosarcoma cell line (kindly provided by Dr. Galietta, Istituto Giannina Gaslini, Genoa, Italy; [22]; alternative splicing isoform of exon 4 encoding a protein of 898 aa; XP_005252878.2) was subcloned into the vector pFROG [39] for heterologous expression (referred to as TMEM16E_898_) and an enhanced green fluorescent protein (EGFP) tag was attached to the TMEM16E C-terminus (referred to as TMEM16E_898_-EGFP). Full-length human TMEM16E (913 aa, UniProt: Q75V66; [36], subcloned into the pCR8/GW/TOPO vector, was kindly provided by Dr. Tobiumi (Hiroshima University, Japan) and carried an EGFP tag at its C-terminus as well ([35]; referred to as TMEM16E_913_-EGFP).

The retinal isoform of mouse TMEM16B [4, 7, 8, 27], subcloned into pCMV-Sport6 (ImaGenes GmbH; NP_705817.1), was used and an EGFP tag was attached to its C-terminus (referred to as TMEM16B-EGFP). Mouse TMEM16F carrying an EGFP tag, after subcloning into the expression vector pEGFP-N1, was kindly provided by Dr. L. Jan (Howard Hughes Medical Institute, San Francisco, USA; [40]; referred to as TMEM16F-EGFP).

PCR-based mutagenesis using the QuikChange XL site-directed mutagenesis kit (Agilent Technologies Italia) was performed to introduce the amino acid substitutions into TMEM16E_898_-EGFP and TMEM16B-EGFP and confirmed by subsequent DNA sequencing of the coding region.

Transient transfection of human embryonic kidney HEK293T and chinese hamster ovary (CHO) cells was done using Effectene reagent (Qiagen, Milan) and 400 ng of plasmid DNA. For expression of TMEM16E_898_, cells were co-transfected with 50 ng pcDNA3.1-E^2^GFP/DsRed plasmid DNA [3] for fluorescence identification of transfected cells.

### Confocal fluorescence microscopy

Transiently transfected HEK293 cells were seeded in glass-bottom petri dishes (custom-made or purchased from IBL Baustoff + Labor GmbH, Austria). Live cell imaging was performed using a Leica TCS-SL confocal laser scanning microscope equipped with 40× or 63× oil immersion objectives (numerical aperture 1.25 and 1.45, respectively). Final images are the average of 4–12 acquisitions. No filtering was applied.

Endoplasmic reticulum was stained using CellLight ER-RFP BacMam 2.0 (Thermo Fisher Scientific), applied to the cell dish 36 h before visualization. The PM marker FM4-64 (Thermo Fisher Scientific) was added at a final concentration of 10 μM in cold solution, and cells were imaged immediately.

### Patch-clamp electrophysiology

Current recordings were performed in the whole-cell patch-clamp configuration between 48 and 96 h after transfection. Patch pipettes were made of borosilicate glass (Hilgenberg, Malsfeld, Germany or Harvard Bioscience) and had resistances of 3–5 MΩ. Currents were recorded with Axopatch 200 or Axopatch 200B amplifiers (Molecular Devices, Sunnyvale, USA) controlled by the custom acquisition program GePulse (by Dr. Michael Pusch, Institute of Biophysics, CNR, Genoa, Italy; freely available at http://users.ge.ibf.cnr.it/pusch/programs-mik.html). Experiments were performed at room temperature (20–24 °C). In experiments requiring solution exchange, the bath was grounded via a 1 M NaCl–agar salt bridge connected to a Ag/AgCl reference electrode. Applied voltages were not corrected for liquid junction potentials.

The extracellular solution contained (in mM): 140 NaCl, 5 K-gluconate, 2 CaSO_4_, 2 MgSO_4_, 10 HEPES, pH 7.4. 10–30 mM glucose was added to reduce volume-regulated chloride currents. In selectivity experiments, the NaCl concentration was reduced to 10 mM and osmolarity was adjusted adding sucrose or replacing NaCl by Na-gluconate. Additionally, 140 NaCl was replaced by equimolar NMDG-Cl. Intracellular solutions contained (in mM): 130 CsCl, 10 HEPES, 10 HEDTA, pH 7.2, and various amounts of CaCl_2_, as calculated with the program WinMAXC [24], to obtain calculated free Ca^2+^ concentrations in the range between 1 and 240 μM. The zero Ca^2+^ solution contained 130 CsCl, 10 HEPES, 2 EGTA, 2 MgCl_2_, pH 7.3, resulting in an estimated free [Ca^2+^] in the low picomolar range. If not otherwise specified, the standard intracellular solution contained 3 µM free Ca^2+^ (with 3.209 mM CaCl_2_ added). All chemicals were purchased from Sigma-Aldrich-Merck (Milano, Italy).

The standard IV stimulation protocol consisted of voltage steps of 300 ms duration ranging from − 100 to + 180 mV or to + 140 mV (with 20-mV increments), followed by a 175-ms tail pulse to − 80 mV, from a holding potential of 0 mV. Current amplitudes were evaluated at the end of the test pulse, between 280 and 300 ms. Other stimulation protocols are given in the figure legends.

Current activation was evaluated from the first potential step at which time-dependent currents were observed (*V*_threshold_). Instantaneous current amplitudes, measured in the time interval between 5 and 10 ms after the onset of the test pulse, were subtracted from the current amplitudes determined at the end of the test pulse (in the time interval between 275 and 300 ms). *V*_threshold_ was defined as the first potential step at which the difference current amplitude exceeded the noise level (standard deviation calculated at the end of the test pulse) of the current traces by more than two times.

To estimate reversal potentials (*V*_rev_), cells were subjected to an activating 200-ms prepulse to + 140 mV, followed by hyperpolarizing steps with 10-mV increments. Tail currents were fitted to single-exponential functions to extrapolate the tail current value at each voltage step. Tail current values were plotted as a function of the applied membrane potential, and *V*_rev_ was estimated from a linear fit in a ± 40-mV interval around *V*_rev_.

Data on the Ca^2+^ dependence of current activation were fitted with the modified Hill equation:

$$I = I_{\text{base}} + \left( {I_{ \text{max} } - I_{\text{base}} } \right)\big/\left[1 + \left( {\left[ {{\text{Ca}}^{2 + } } \right]\Big/\left[ {{\text{Ca}}^{2 + } } \right]_{0.5} } \right)^{h} \right],$$

where [Ca^2+^]_0.5_ is the half-maximal Ca^2+^ concentration and *h* is the Hill coefficient.

Data analysis and figures were made using Ana (freely available at http://users.ge.ibf.cnr.it/pusch/programs-mik.htm) and Igor Pro software (Wavemetrics, Lake Oswego, OR, USA). For the sake of clarity, the capacitative transients of some current traces were trimmed in the figures.

### Phospholipid scrambling assay

Transiently transfected HEK293 cells were seeded in glass-bottom petri dishes and tested for scramblase activity after 48–72 h (TMEM16E_898_-EGFP, TMEM16F-EGFP) or 36–48 h (TMEM16E_898_^T498I^-EGFP). Cells were washed in a buffer solution (140 mM NaCl, 2.5 or 5 mM CaCl_2_, 10 mM HEPES, pH 7.4) and incubated with Cy3-conjugated annexin-V (Enzo Life Sciences), at a dilution of 1:100–200, in the absence or presence of the Ca^2+^-ionophore A23187 (5–10 μM; Sigma-Aldrich-Merck) in cold solution for 5 min. Ionophore solution was prepared freshly from a 1-mM stock solution (in DMSO) stored at − 20 °C.

In live cell experiments, fresh buffer solution was added to the petri dish after the incubation period. Alternatively, cells were washed with buffer solution and fixed for 5 min at room temperature by adding formalin solution (10%). After further washing, cells were observed by fluorescence microscopy using a Leica TCS-SL confocal microscope (see “Confocal fluorescence microscopy”).

Ca^2+^-dependent activation of scrambling activity was further assessed by measuring annexin-V binding during patch-clamp experiments in HEK293 expressing TMEM16E_898_-EGFP and in non-transfected control cells. Cells were bathed in standard extracellular solution (in mM: 140 NaCl, 5 KCl, 2 CaCl_2_, 1 MgCl_2_, 10 glucose, 10 HEPES, pH 7.4) supplemented with Alexa Fluor 555-conjugated annexin-V (Life Technologies Italia), and scramblase activity was stimulated using standard intracellular solution containing 3 μM free Ca^2+^. To avoid photobleaching, fresh annexin-V was added after cell selection. Alexa Fluor 555 was excited at 555 nm using a polychromatic light selector equipped with a Xenon lamp (Polychrome V, Till Photonics) and fluorescence signals were acquired using an Imago CCD camera (TILL Photonics) mounted on a Zeiss Axiovert 200 inverted microscope equipped with a 100× oil objective (1.3 numerical aperture). After the whole-cell configuration was achieved, time-lapse imaging was performed at 1-min intervals synchronously with voltage-clamp recordings (IV protocols in 5-min intervals).

TILLvisION imaging software (TILL Photonics) was used for data acquisition, while ImageJ (NIH, Bethesda, MD, [30]) and Igor Pro for data analysis. Data are shown as the integrated fluorescence intensity of a region of interest that included the whole cell, normalized to the average of the maximal fluorescence change observed in transfected HEK cells within a 25-min acquisition period. The baseline fluorescence intensity measured at the beginning of the recording period was subtracted for each cell.

### Statistical analysis

Data are presented as mean ± sem, with n indicating the number of cells. Normality of the data was assessed using the Shapiro–Wilk test. Statistical significance was determined using paired *t* test or Mann–Whitney *U* test. *P* values < 0.05 were considered significant.

### Electronic supplementary material

Below is the link to the electronic supplementary material.

Supplementary material 1. (PDF 763 kb)

## Abbreviations

PM: Plasma membrane
PLS: Phospholipid scrambling
