# Supplementary material for: Gain of function of TMEM16E/ANO5 scrambling activity caused by a mutation associated with gnathodiaphyseal dysplasia
Source: Cell Mol Life Sci. 2017 Nov 9;75(9):1657–70. doi: 10.1007/s00018-017-2704-9 (PMC5897490; doi:10.1007/s00018-017-2704-9)
Supplement: Supplementary file 1 — Supplementary material 1. (PDF 763 kb) [file 18_2017_2704_MOESM1_ESM.pdf]

Supplementary Figures

S1

```
hTMEM16E898    ---MGDPDLLEVL-----AEEGEKVNKHIDYSFQMSE-SLSSRETSFLINEET-------   44
hTMEM16E913    ---MGDPDLLEVL-----AEEGEKVNKHIDYSFQMSEQSLSSRETSFLINEETMPAKRFN   52
mTMEM16F       MQMMTRKVLLNMELEEDDDEDGDIVLENFDQ-------TIVCPTFGSLENQQDF------   47
mTMEM16B       ------------------------------------------------------------   0
mTMEM16A(ac)   ---MRVPEKYSTLPAE----DRSV---HIVN-------ICAIEDLGYLPSEGT-------   36

hTMEM16E898    -------MFQKNQQSKDSIFFRDGIRQIDFVLSYVDDVKKD-------------------   78
hTMEM16E913    LFLRRRLMFQKNQQSKDSIFFRDGIRQIDFVLSYVDDVKKD-------------------   93
mTMEM16F       ----RTPEFEEFNGKPDSLFFTDGQRRIDFILVYEDESKKE-------------------   84
mTMEM16B       ------------------MHFHDNQRKVDYVLAYHYRKRGAHLGHGSPGHSLAVISNG-E   41
mTMEM16A(ac)   --LLNSLSVDPDAECKYGLYFRDGKRKVDYILVYHHKRASGSRTLARRGLQNDMVLGTRS   94

hTMEM16E898    --------------------AELKA------ERRKEFETNLRKTGLELEIEDKRDSEDGR   112
hTMEM16E913    --------------------AELKA------ERRKEFETNLRKTGLELEIEDKRDSEDGR   127
mTMEM16F       --------------------NNKKGTNEKQKRKRQAYESNLICHGLQL--EATRSVSDDK   122
mTMEM16B       TGKER---HGGGPG--DVELGPLDALEEERREQRDEFEHNLMAAGLELE--KDLESKSQG   94
mTMEM16A(ac)   VRQDQPLPGKGSPVDAGSPEVPMDYHEDDKRFRREEYEGNLLEAGLELE--NDEDTKIHG   152

hTMEM16E898    TYFVKIHAPWEVLVTYAEVLGIKMPIKESDIPRP---KHTPISYVLGPVRLPLSVKYPH-   168
hTMEM16E913    TYFVKIHAPWEVLVTYAEVLGIKMPIKESDIPRP---KHTPISYVLGPVRLPLSVKYPH-   183
mTMEM16F       LVFVKVHAPWEVLCTYAEIMHIKLPLKPNDLKTR--SPFGNLNWFTKVLRVNESVIKPE-   179
mTMEM16B       SVFVRIHAPWQVLAREAEFLKIKVPTKKMYEIKAGGSIAKKFSAILQTLSSPLQPRVPEH   154
mTMEM16A(ac)   VGFVKIHAPWHVLCREAEFLKLKMPTKKVYHISETRGLLKTINSVLQKITDPIQPKVAEH   212

hTMEM16E898    ----PEYFTAQFSRHRQELFLIEDQATFFPSSSRNRIVYYILSRCPFGIEDGKKRFGIER   224
hTMEM16E913    ----PEYFTAQFSRHRQELFLIEDQATFFPSSSRNRIVYYILSRCPFGIEDGKKRFGIER   239
mTMEM16F       ----QEFFTAPFEKSRMNDFYILDRDSFFNPATRSRIVYFILSRVKYQVMNNVNKFGINR   235
mTMEM16B       SNNRMKNLSYPFSREKMYLYNIQEKDTFFDNATRSRIVHEILKRTACS--RANNTMGINS   212
mTMEM16A(ac)   RPQTTKRLSYPFSREKQHLFDLTDRDSFFDSKTRSTIVYEILKRTTCT--KAKYSMGITS   270

hTMEM16E898    LLNSNTYSSAYPLHDGQYWKPSEPPNPTNERYTLHQNWARFSYFYKEQPLDLIKNYYGEK   284
hTMEM16E913    LLNSNTYSSAYPLHDGQYWKPSEPPNPTNERYTLHQNWARFSYFYKEQPLDLIKNYYGEK   299
mTMEM16F       LVSSGIYKAAFPLHDCRFNYESEDISCPSERYLLYREWAHPRSIYKKQPLDLIRKYYGEK   295
mTMEM16B       LIANNIYEAAYPLHDGEYDSPGDD---MNDRKLLYQEWARYGVFYKFQPIDLIRKYFGEK   269
mTMEM16A(ac)   LLANGVYSAAYPLHDGDYEGDNVE---FNDRKLLYEEWASYGVFYKYQPIDLVRKYFGEK   327

hTMEM16E898    IGIYFVFLGFYTEMLFFAAVVGLACFIYGLLSMEHNTSSTEICDPEIGGQMIMCPLCDQV   344
hTMEM16E913    IGIYFVFLGFYTEMLFFAAVVGLACFIYGLLSMEHNTSSTEICDPEIGGQMIMCPLCDQV   359
mTMEM16F       IGIYFAWLGYYTQMLLLAAVVGVACFLYGYLDQDNCTWSKEVCDPDIGGQILMCPQCDRL   355
mTMEM16B       IGLYFAWLGLYTSFLIPSSVIGVIVFLYGCATIEEDIPSKEMCDHQ--NAFTMCPLCDKS   327
mTMEM16A(ac)   VGLYFAWLGAYTQMLIPASIVGVIVFLYGCATVDENIPSMEMCDQR--YNITMCPLCDKT   385
                          TM1

hTMEM16E898    CDYWRLNSTCLASKFSHLFDNESTVFFAIFMGIWVTLFLEFWKQRQARLEYEWDLVDFEE   404
hTMEM16E913    CDYWRLNSTCLASKFSHLFDNESTVFFAIFMGIWVTLFLEFWKQRQARLEYEWDLVDFEE   419
mTMEM16F       CPFWRLNITCESSKKLCIFDSFGTLIFAVFMGVWVTLFLEFWKRRQAELEYEWDTVELQQ   415
mTMEM16B       CDYWNLSSACGTARASHLFDNPATVFFSIFMALWATMFLENWKRLQMRLGYFWDLTGIEE   387
mTMEM16A(ac)   CSYWKMSSACATARASHLFDNPATVFFSVFMALWAATFMEHWKRKQMRLNYRWDLTGFEE   445
                                                   TM2

hTMEM16E898    EQQ--QLQLRPEFEAMCKHRKLNAVT--------------KEMEPYMP-LYTRIPWYFLS   447
hTMEM16E913    EQQ--QLQLRPEFEAMCKHRKLNAVT--------------KEMEPYMP-LYTRIPWYFLS   462
mTMEM16F       E-----EQARPEYEAQCNHVVINEIT--------------QEEERIPFTTCGKCIRVTLC   456
mTMEM16B       EEERSQEHSRPEYETKVREKLLKESGKSAVQKLEANSPEDDEDDEDKLTWKDRFPGYLMN   447
mTMEM16A(ac)   EEEAVKDHPRAEYEARVLEKSLRKES------------RNKETDKVKLTWRDRFPAYFTN   493
                                                                       TM3
```

S1

```
 hTMEM16E898   GATVTLWMSLVVTSMVAVIVYRLSVFATFASFMESD-ASLKQVKSFLTPQITTSLTGSCL  506
 hTMEM16E913   GATVTLWMSLVVTSMVAVIVYRLSVFATFASFMESD-ASLKQVKSFLTPQITTSLTGSCL  521
    mTMEM16F   ASAVFFWILLIIASVIGIIVYRLSVFIVFSTTLPKNPNGTDPIQKYLTPQMATSITASII  516
    mTMEM16B   FASILFMIALTFSIVFGVIVYRITTAAALSL--NK--ATRSN------VRVTVTATAVII  497
mTMEM16A(ac)   LVSIIFMIAVTFAIVLGVIIYRISTAAALAM--NSSPSVRSN------IRVTVTATAVII  545
                              TM3                                     TM4

 hTMEM16E898   NFIVILILNFFYEKISAWITKMEIPRTYQEYESSLTLKMFLFQFVNFYSSCFYVAFFKGK  566
 hTMEM16E913   NFIVILILNFFYEKISAWITKMEIPRTYQEYESSLTLKMFLFQFVNFYSSCFYVAFFKGK  581
    mTMEM16F   SFIIIMILNTIYEKVAIMITNFELPRTQTDYENSLTMKMFLFQFVNYYSSCFYIAFFKGK  576
    mTMEM16B   NLVVILILDEIYGAVAKWLTKIEVPKTEQTFEERLILKAFLLKFVNAYSPIFYVAFFKGR  557
mTMEM16A(ac)   NLVVIILLDEVYGCIARWLTKIEVPKTEKSFEERLTFKAFLLKFVNSYTPIFYVAFFKGR  605
                              TM4                            TM5

 hTMEM16E898   FVGYPGKYTYLFNEWRSEECDPGGCLIELTTQLTIIMTGKQIF-GNIKEAIYPLALNWWR  625
 hTMEM16E913   FVGYPGKYTYLFNEWRSEECDPGGCLIELTTQLTIIMTGKQIF-GNIKEAIYPLALNWWR  640
    mTMEM16F   FVGYPGDPVYLLGKYRSEECDPGGCLLELTTQLTIIMGGKAIW-NNIQEVLLPWVMNLIG  635
    mTMEM16B   FVGRPGSYVYVFDGYRMEECAPGGCLMELCIQLSIIMLGKQLIQNNIFEIGVPKLKKLFR  617
mTMEM16A(ac)   FVGRPGDYVYIFRSFRMEECAPGGCLMELCIQLSIIMLGKQLIQNNLFEIGIPKMKKFIR  665
                                                             TM6

 hTMEM16E898   RRKARTN-------SEKLYSRWEQDHDLESFGPLGLFYEYLETVTQFGFVTLFVASFPLA  678
 hTMEM16E913   RRKARTN-------SEKLYSRWEQDHDLESFGPLGLFYEYLETVTQFGFVTLFVASFPLA  693
    mTMEM16F   RYKRVSG-------SEKITPRWEQDYHLQPMGKLGLFYEYLEMIIQFGFVTLFVASFPLA  688
    mTMEM16B   KLKDETEPGESDPDHSKRPEQWDLDHSLEPYT--GLTPEYMEMIIQFGFVTLFVASFPLA  675
mTMEM16A(ac)   YLKLRRQSPSDREEYVKRKQRYEVDFNLEPFA--GLTPEYMEMIIQFGFVTLFVASFPLA  723
                                                              TM7

 hTMEM16E898   PLLALINNIVEIRVDAWKLTTQYRRTVASKAHSIGVWQDILYGMAVLSVATNAFIVAFTS  738
 hTMEM16E913   PLLALINNIVEIRVDAWKLTTQYRRTVASKAHSIGVWQDILYGMAVLSVATNAFIVAFTS  753
    mTMEM16F   PLLALVNNILEIRVDAWKLTTQFRRMVPEKAQDIGAWQPIMQGIAILAVVTNAMIIAFTS  748
    mTMEM16B   PVFALLNNVIEVRLDAKKFVTELRRPDAVRTKDIGIWFDILSGIGKFSVIINAFVIAVTS  735
mTMEM16A(ac)   PLFALLNNIIEIRLDAKKFVTELRRPVAIRAKDIGIWYNILRGVGKLAVIINAFVISFTS  783
                            TM8                               TM9

 hTMEM16E898   DIIPRLVYYYAYSTN-----ATQPMTGYVNNSLSVFLIADFPNHTAPS---EK-RDFITC  789
 hTMEM16E913   DIIPRLVYYYAYSTN-----ATQPMTGYVNNSLSVFLIADFPNHTAPS---EK-RDFITC  804
    mTMEM16F   DMIPRLVYYWSFSIPPYGDHTYYTMDGYINNTLSVFNITDFKNTDKENPYIGL-GNYTLC  807
    mTMEM16B   DFIPRLVYQYSYSHN-------GTLHGFVNHTLSFFNVSQLKEGTQPENS-QFDQEVQFC  787
mTMEM16A(ac)   DFIPRLVYLYMYSQN-------GTMHGFVNHTLSSFNVSDFQNGTAPNDPLDLGYEVQIC  836

 hTMEM16E898   RYRDYRYPPDDENKYFHNMQFWHVLAAKMTFIIVMEHVVFLVKFLLAWMIPDVPKDVVER  849
 hTMEM16E913   RYRDYRYPPDDENKYFHNMQFWHVLAAKMTFIIVMEHVVFLVKFLLAWMIPDVPKDVVER  864
    mTMEM16F   RYRDFRNPPGHPQEYKHNIYYWHVIAAKLAFIIVMEHIIYSVKFFISYAIPDVSKITKSK  867
    mTMEM16B   RFKDYREPPWAPNPYEFSKQYWSVLSARLAFVIIFQNLVMFLSVLVDWMIPDIPTDISDQ  847
mTMEM16A(ac)   RYKDYREPPWSEHKYDISKDFWAVLAARLAFVIVFQNLVMFMSDFVDWVIPDIPKDISQQ  896
                                                TM10

 hTMEM16E898   IKREKLMTIKILHDFELNKLKENLGINSNEFAKHVMIEENKAQLAK----STL-------  898
 hTMEM16E913   IKREKLMTIKILHDFELNKLKENLGINSNEFAKHVMIEENKAQLAK----STL-------  913
    mTMEM16F   IKREKYLTQKLLHESHLKDLTKNMGIIAERIGGTVDNSV-RPKLE---------------  911
    mTMEM16B   IKKEKSLLVDFFLKEEHEKVKLADEPTQRSQGGGDRSRRSRAASSAPSGRSQPGSIASSG  907
mTMEM16A(ac)   IHKEKVLMVELFMREEQGKQQLLDTWMEKEKPRDVPCNNHSPTTHPEAGDGSP---VPSY  953

 hTMEM16E898   -------                                                       898
 hTMEM16E913   -------                                                       913
    mTMEM16F   -------                                                       911
    mTMEM16B   SQHTNV-                                                       913
mTMEM16A(ac)   EYHGDAL                                                       960
```

S2

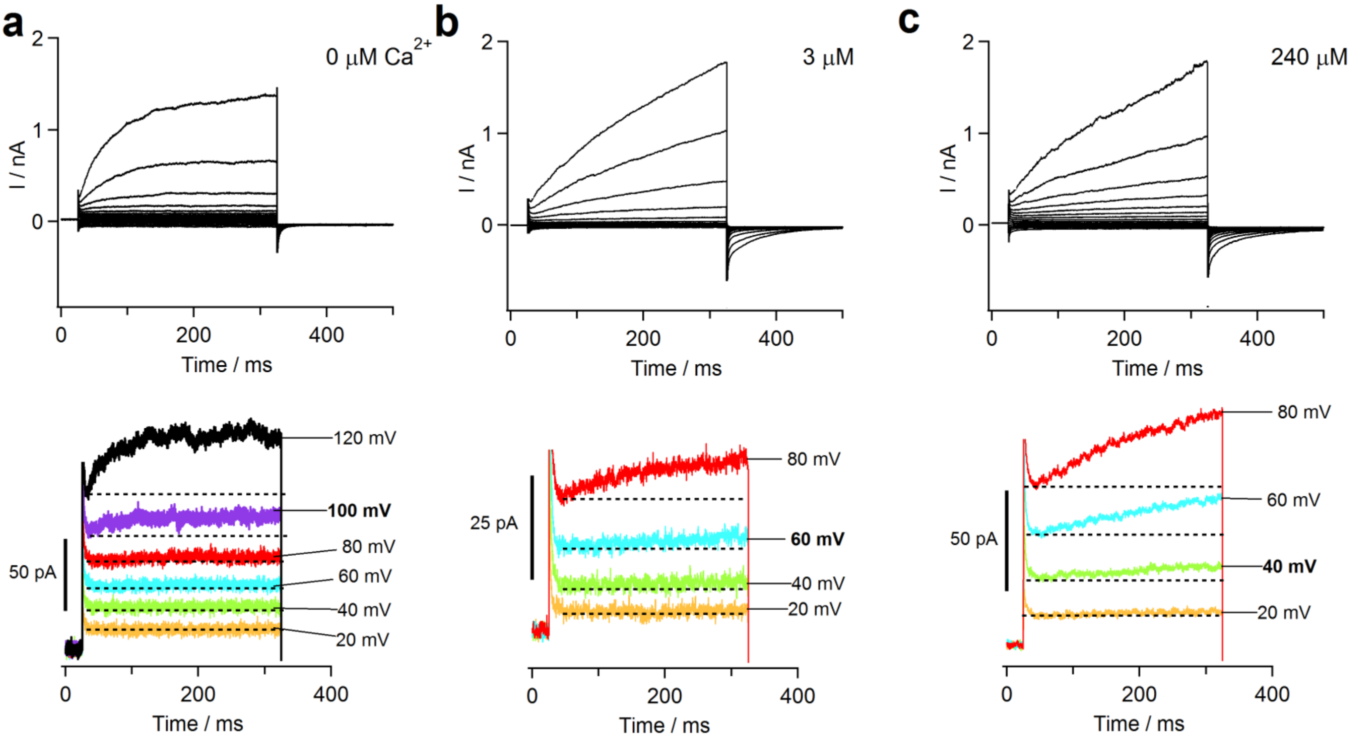
a
b
c
0 μM Ca2+
3 μM
240 μM
I / nA
Time / ms
120 mV
100 mV
80 mV
60 mV
40 mV
20 mV
50 pA
25 pA

S3

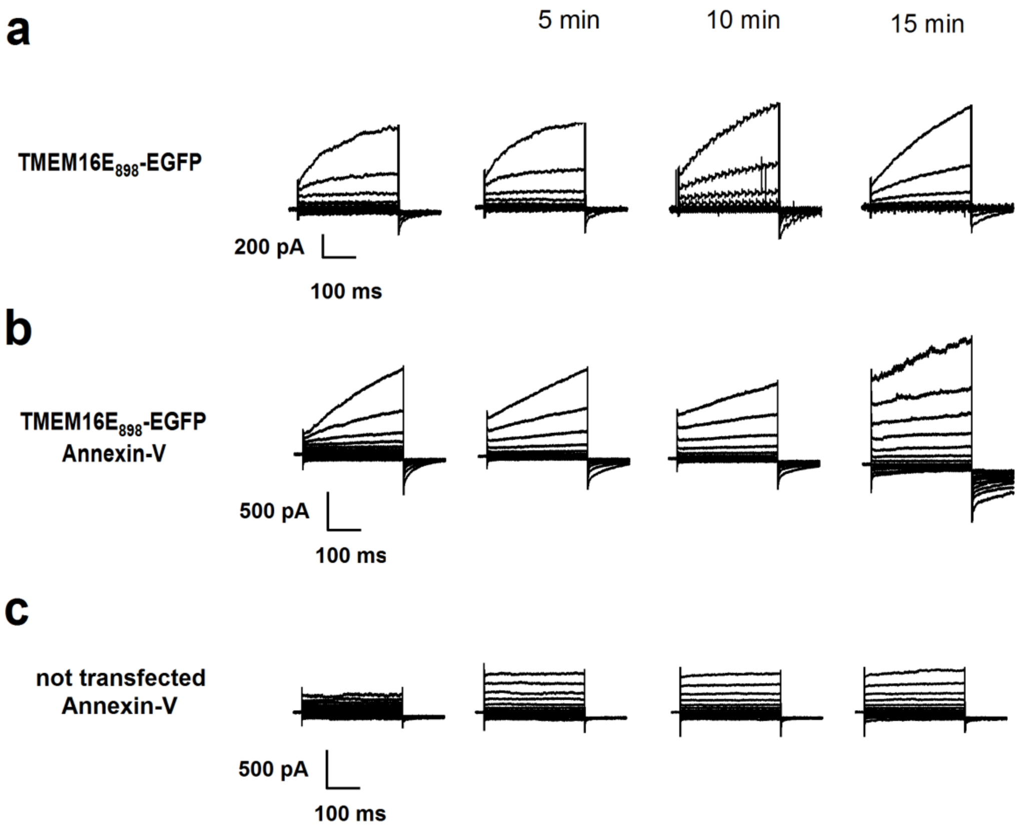

a
5 min
10 min
15 min
TMEM16E898-EGFP
200 pA
100 ms
b
TMEM16E898-EGFP
Annexin-V
500 pA
100 ms
c
not transfected
Annexin-V
500 pA
100 ms

LEGENDS

FIGURE S1: Sequence alignment of the TMEM16 proteins used in this study.

Protein sequence alignment of the two human TMEM16E isoforms, mouse TMEM16F, mouse TMEM16B and mouse TMEM16A (isoform ac). Predicted trans-membrane domains, based on nhTMEM16 and mTMEM16A protein structures (Brunner et al., 2014; Paulino et al., 2017), are shown in grey. Extracellular and intracellular amino acid residues are highlighted in blue and green, respectively. Residues of the putative scrambling domain in TMEM16E and TMEM16F (Gyobu et al., 2015; Yu et al., 2015) are indicated in bold. The position of the GDD-related T498I exchange in TMEM16E is highlighted in red. Alignment was performed using CLUSTAL OMEGA (1.2.4) (Web Services of EMBL-EBI).

FIGURE S2: $V_{threshold}$ of TMEM16E currents shifts negative with increasing cytosolic Ca^{2+} concentrations.

Whole-cell patch-clamp recordings in CHO cells transfected with $TMEM16E_{898}$-EGFP, using pipette solutions containing zero Ca^{2+} (**a**), 3 µM free Ca^{2+} (**b**) and 240 µM free Ca^{2+} (**c**). *Upper panel*, current traces elicited by voltage steps ranging from -100 to +180 mV with 20-mV increments. *Lower panel*, same recordings shown at smaller amplitude scales, in a voltage range close to the threshold potential of current activation (*value in bold*).

In order to illustrate the Ca^{2+} dependence of TMEM16E current activation, recordings with approximately comparable current amplitudes are shown. Note that these recordings are not representative of the mean current amplitudes determined at the respective Ca^{2+} concentration (**Figure 4**).

FIGURE S3: Time-dependent modification of membrane currents in combined patch-clamp/ PLS experiments.

Whole-cell patch-clamp recordings in response to repeated voltage stimulation (-100 to +180 mV with 20-mV increments) at the indicated time points, using a pipette solution containing 3 µM free Ca^{2+}: (**a**) HEK293 cell transfected with $TMEM16E_{898}$-EGFP in standard bath solution, (**b**) HEK293 cell transfected with $TMEM16E_{898}$-EGFP in bath solution supplemented with Alexa Fluor 555-conjugated annexin-V, (**c**) non-transfected HEK293 cell in bath solution supplemented with Alexa Fluor 555-conjugated annexin-V.
